# Effects of Curtailed Juvenile State on Cardiac Structure and Function in Adulthood: The Fels Longitudinal Study

**DOI:** 10.21767/2572-5394.100018

**Published:** 2016-10-06

**Authors:** Nak-Kyeong Kim, Roy T Sabo, Aobo Wang, Cynthia S Sabo, Shumei S Sun

**Affiliations:** Department of Biostatistics, School of Medicine, Virginia Commonwealth University, Richmond, Virginia, USA

**Keywords:** Curtailed juvenile state, Cardiac measurement, BMI, Obesity, Linear mixed effect model, Repeated measure

## Abstract

**Objective:**

Previous studies have shown associations between body mass index and cardiac structure in both childhood and adulthood. Using Fels Longitudinal Study measurements, we investigate the relationships between a curtailed juvenile state and both adult cardiac structure and function.

**Methods:**

A linear mixed-effect repeated measure analysis of variance model is used to test if there is a relationship between juvenile state and each echocardiographic measurement.

**Results:**

The curtailed juvenile state is significantly associated with adult left ventricular mass index for both males and females. It is also significantly associated with the interventricular septal wall thickness index and relative wall thickness index for females. In both cases, early juvenile states led to more abnormal structural estimates in adulthood than did late juvenile states. Among cardiac function measurements such as left ventricular ejection fraction and left ventricular shortening fraction, left ventricular ejection fraction is significantly associated with the juvenile state for females.

**Conclusion:**

The curtailed juvenile state at the childhood may have a long-term adverse effect on adult cardiac structure and function abnormalities.

## Background

Cardiac structure measurements (like left ventricular mass, or LVM) have been shown to independently predict the incidence of several events attributable to cardiovascular disease, including fatality [1]. In turn, body mass index (BMI) has been shown to be associated with adverse cardiac structure [2–7], while measurements of obesity, adiposity and blood pressure have specifically been found to be associated with both left ventricular mass (LVM) [8–16] and relative wall thickness (RWT) [17]. Cardiac function measurements (like left ventricular shortening fraction or ejection fraction) have also been found associated with contemporaneous BMI [18] and blood pressure [18,19]. These findings imply a deep and possibly complex interaction between body size, adiposity, cardiac structure and function, and cardiovascular health outcomes.

In light of this interconnection, there is growing evidence that the effects of body size, composition, and blood pressure on adult cardiac structure begin in childhood, with childhood BMI in particular having a significant impact on cardiac structure [20–26]. Two studies from the Bogalusa Heart Study (BHS) found that childhood BMI was significantly associated with abnormal LVM in adulthood [25,26]. Sabo et al. found relationships between childhood BMI and LVM index (in males and females) and interventricular septal thickness (in males), and also between waist circumference (indexed by height) and LVM index in males, with associations appearing as early as 8 years [27]. Investigating the childhood origins of abnormal cardiac structure and function have important clinical and public health consequences, as corrective interventions can be targeted in early childhood or adolescence to avoid abnormal health in adulthood. The literature on associations between childhood anthropomorphic measurements and adult cardiac function is sparse.

Guo et al. showed that in addition to childhood body size, the timing of body size change can also affect adult cardiovascular health [28]. The BMI or adiposity rebound is a childhood growth milestone, defined as the age at which early childhood BMI stops decreasing and begins increasing, usually between ages 4 and 6. Several studies have shown that the BMI rebound is related to several adulthood cardiovascular measures and disease [28–31]. In this study, we investigate the effect of a curtailed juvenile state, defined as an early attainment of BMI rebound, on measurements of cardiac structure and function in adulthood. Predicting adult cardiac structure by observing a curtailed juvenile state has important clinical and public health implications by suggesting that early interventions can be developed to improve cardiovascular health.

Using serial childhood measurements from the Fels Longitudinal Study (FLS), we classify three juvenile states (curtailed, normal, and prolonged) based on the first and third quartiles of age at BMI rebound. We then investigate associations between these juvenile state classifications on adult cardiac structure and function measurements from the FLS datasets, such as left ventricular mass index (LVMI), interventricular septal wall thickness index (IVSTI), relative wall thickness index (RWTI), left ventricular ejection fraction (LVEF), and left ventricular shortening fraction (LVSF).

## Methods

### Data source and participants

This study consisted of a subset of FLS consists of predominantly white Americans who were selected to undergo echocardiographic examinations. The FLS has continuously enrolled participants since 1929, generally at birth, and does not select participants with respect to factors known to be associated with body composition, cardiac health, and other related conditions. Participants are examined semi-annually from birth until 18 years, and are examined every other year thereafter. More information on the FLS and its participants can be found elsewhere [32,33]. Written informed consent for study participation was provided either directly from the individual or their parents, and all procedures were approved by the Institutional Review Boards at Wright State University and Virginia Commonwealth University.

A total of 815 participants aged between 2 and 25 years (412 males and 403 females) who have at least 10 serial examinations were selected. For each participant, a cubic polynomial of age was fitted to BMI as a response variable. The timing of the BMI rebound is defined as the age that minimizes the fitted cubic BMI curve, indicating the onset of the juvenile state for each participant. Individuals with a poor fit (RMSE>1.5) and individuals with no cardiac structure and function measurements were removed. The remaining participants included 342 subjects (162 males and 180 females).

### Measurements

Height and weight were measured according to recommendations in the Anthropometric Standardization Reference Manual [34], with stature measured to 0.1 cm using a Holtain stadiometer, weight measured to 0.1 kg using a SECA scale, and body mass index (BMI, kg/m^2^) calculated as the ratio of weight to height-squared (in meters). Both measurements were taken twice, with a third taken if the difference between the two measurements exceeds established tolerance (0.3 kg for weight; 0.5 cm for height). The average of all values were used for analysis.

The echocardiographic measurements were taken by a certified sonographer using an ATL Philips Medical System HDI 5000 ultrasound imaging system. Two-dimensional and two-dimensional directed M-mode echocardiographic images were recorded, and following the American Society of Echocardiography (ASE) recommendation, measurements were made on three or more cardiac cycles. Left ventricular mass (LVM) was calculated using the ASE formula: LVM=0.8(1.04([LVIDd+PWTd+IVST]^3^-[LVIDd]^3^))+0.6 g, where LVIDd is left ventricular internal dimension at end diastole, PWTd is posterior wall thickness at end diastole, and IVST is interventricular septal wall thickness at end diastole. Relative wall thickness (RWT) was calculated as RWT=2(PWTd)/(LVIDd). Left ventricular ejection fraction was calculated as: LVEF = (LVEDV − LVESV)/LVEDV, where LVEDV is the end diastolic left ventricular volume and LVESV is the end systolic left ventricular volume. Left ventricular shortening fraction was calculated as: LVSF = (LVEDd − LVESd)/LVEDd, where LVEDd is the end diastolic left ventricular dimension and LVESd is the end systolic left ventricular dimension. Since LVM, IVST, and RWT are positively correlated with height [5,24], we adjust for height by dividing these by height raised to the 2.7 power, following a convention [35], resulting LVMI, IVSTI, and RWTI, where the suffix “I” stands for indexed by height.

## Statistical Analysis

A three-degree polynomial model is used to estimate childhood BMI growth curve parameters within each subject [28]. This model regresses childhood BMI values against a three-degree (cubic) polynomial in terms of age $\left(BMI={\hat{\beta }}_{0}+{\hat{\beta }}_{1}Age+{\hat{\beta }}_{2}Age^{2}+{\hat{\beta }}_{3}Age^{3}\right)$. Though several childhood growth parameters can be estimated from this model (including ages and BMI at points of maximum growth velocity and maximum post-pubescent growth attainment), we only estimate the age at adiposity rebound. Since we use a smooth growth curve (a cubic polynomial) to fit the observed BMI values over ages, we can have a unique estimate at adiposity rebound. Specifically, taking the three coefficients from the cubic model ($\left({\hat{\beta }}_{1},{\hat{\beta }}_{2},{\hat{\beta }}_{3}\right)$, age at BMI rebound for each subject is estimated using $\left(-{\hat{\beta }}_{2}+\sqrt{{\hat{\beta }}_{2}^{2}-3{\hat{\beta }}_{1}{\hat{\beta }}_{3}}\right)/3{\hat{\beta }}_{3}$, which is the age for achieving the minimum BMI in the fitted growth curve. From these estimates from all subjects (one estimate per subject) we estimate juvenile state using the first and third quartiles for both males and females. Males are defined as having a curtailed juvenile state (Early) if their BMI rebound estimate is less than the first quartile (4.8), as having a normal juvenile state (Normal) if their BMI rebound estimate is greater than the first quartile and less than the third quartile (6.7), and as having a prolonged juvenile state (Late) if their BMI rebound estimate is greater than the third quartile. Juvenile states for females are defined similarly based on first and third quartiles of 4.6 and 6.6, respectively.

A linear mixed-effect repeated measure analysis of variance (ANOVA) model is used to test if there is a relationship between juvenile state (Early, Normal, and Late) and each echocardiographic measurement. Specifically, the response of this model is one of the adult echocardiographic measurements (LVMI, IVSTI, RWTI, LVEF or LVSF), with fixed effects of juvenile state, adult age (when then echocardiographic measurement was taken), and the interaction between juvenile state and age. The model formula can be expressed as

$$y_{ij}=\mu +\gamma_{1}State.{\left(-1\right)}_{i}+\gamma_{2}State.0_{i}+\gamma_{3}Age_{ij}\;+\gamma_{4}{\left(State\cdot (-1)\times Age\right)}_{ij}+\gamma_{5}{\left(State.0\times Age\right)}_{ij}+\epsilon_{ij},$$

for *i*=1,...,*n* and *j*=1,...,t, where *y*_ij_ is one of the echocardiographic measurements for subject *i* at time *j*, State. (m)*i* is an indicator holding 1 when the juvenile state of subject *i* is m, 0 otherwise, and ε_ij_’s are multivariate normal errors with their covariance within a subject having a compound symmetry structure.

We need two indicators to represent the juvenile state because it has three levels: (−1, 0, 1) = (Early, Normal, Late). Note that these models are fit separately for males and females because they seem to follow different growth curves [36]. A participant-level random effect was included to account for the within-participant dependence, using the compound symmetry covariance structure (since adult measurements were not equally spaced). In this model, we are particularly interested in testing three hypotheses: a difference between the estimated mean echocardiographic measurement (1) between participants with curtailed (Early) and normal (Normal) juvenile states, (2) between participants with Early and prolonged (Late) juvenile states, and (3) between participants with Normal and Late juvenile states. These tests are conducted assuming adult ages of 20, 30, 40 and 50. A 5% significance level was used for all tests.

## Results

Adult measurements are summarized with means, standard deviations, and minimum and maximum values in Table 1. Age for females is greater than age for males, with a mean difference of 2.4 years. Among cardiac structure measurements (LVMI, IVSTI, and RWTI), LVMI is similar between males and females; IVSTI and RWTI are higher on average for females than for males.

The cardiac function measurements (LVEF and LVSF) are comparable between males and females. The model parameter estimates are shown at Table S1 in Supplementary Material, for all five echocardiographic measurements in males and females. As mentioned in the previous section, however, our focus is not on testing the significance of parameter estimates; we are interested in estimated mean differences between juvenile states at ages of 20, 30, 40 and 50.

### Left ventricular mass index

**Figure 1:**
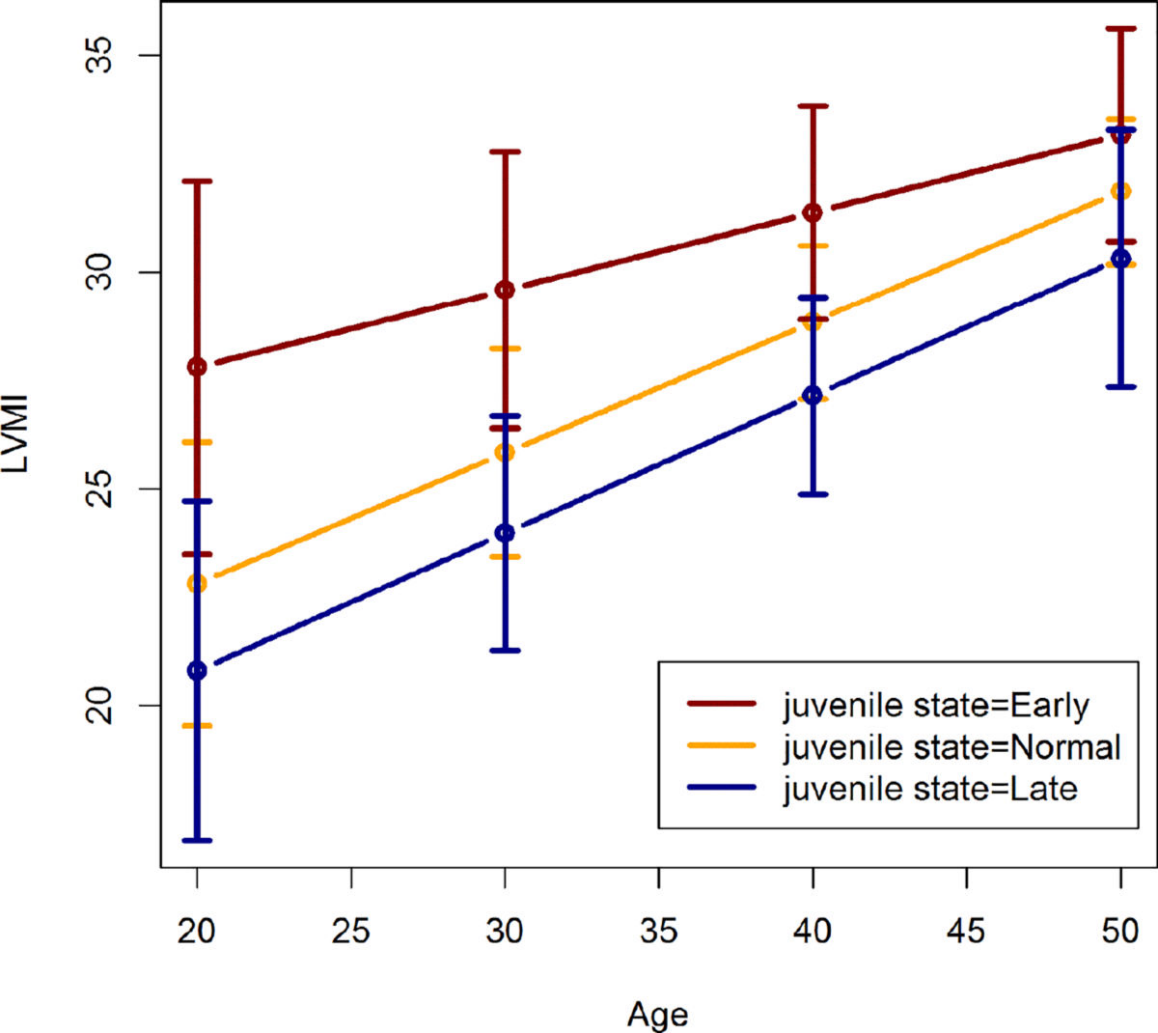
Estimated left ventricular mass index in males by juvenile state.

In males (Table 2 and Figure 1), we see that an Early juvenile state leads to higher estimated LVMI than a Late juvenile state at ages 20 through 40 (p<0.019). The difference in estimated LVMI between these groups of participants decreases as adult age increases, and disappears by age 50 (p=0.147).

There are no significant differences in estimated LVMI between participants with Early and Normal juvenile states, though the LVMI means are uniformly lower throughout adulthood in those with Normal juvenile states.

**Figure 2:**
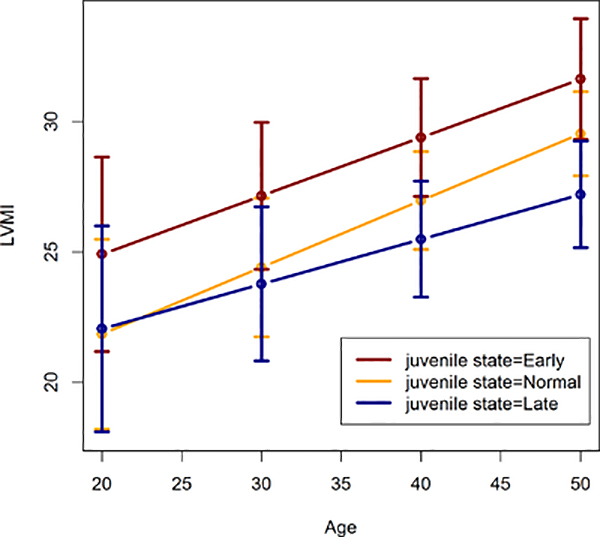
Estimated left ventricular mass index in females by juvenile state.

In females (Table 2 and Figure 2), we see that an Early juvenile state leads to higher estimated LVMI throughout adulthood than does a Late juvenile state, though the estimates are only significantly different at ages 40 and 50 (p<0.017). Estimates for a Normal juvenile state are lower than for an early juvenile state at ages 30 through 50, but not significantly.

### Interventricular septal wall thickness index

**Figure 3:**
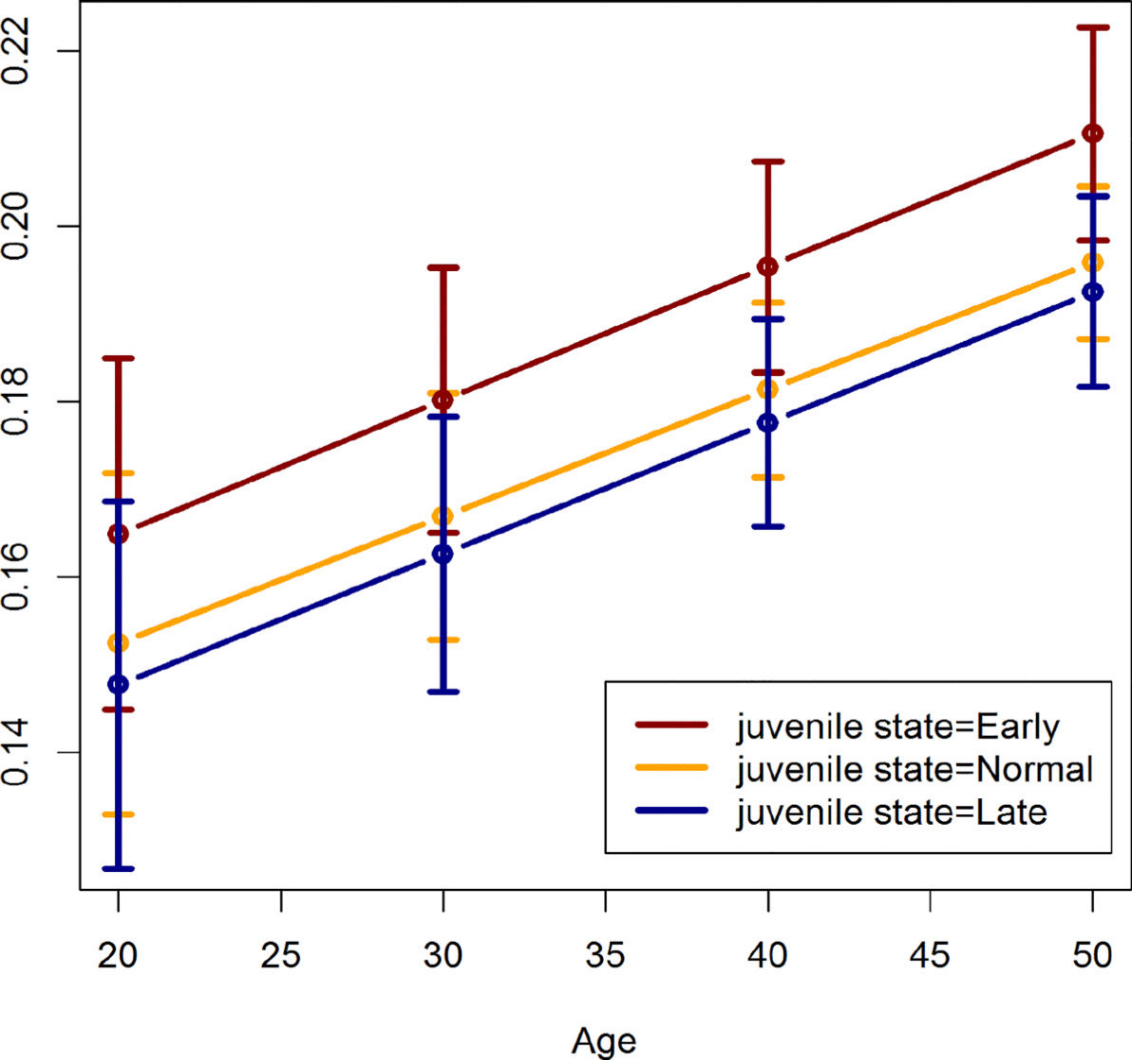
Estimated interventricular septal wall thickness index in females by juvenile state.

For males there are no significant differences in IVSTI between juvenile state classifications at any adult age. For females, the estimated IVSTI values are shown in Table 2 and Figure 3. There is an increasing trend for IVSTI with age.

Though the differences in estimated IVSTI between juvenile state groups are almost constant throughout adulthood, Early juvenile state is only significantly larger than Late juvenile stat at ages 40 and 50 (p<0.040).

Estimated IVSTI values in the Normal juvenile state are similar to those in the late juvenile state, but they are not significantly different from those in the early juvenile state.

### Relative wall thickness index

**Figure 4:**
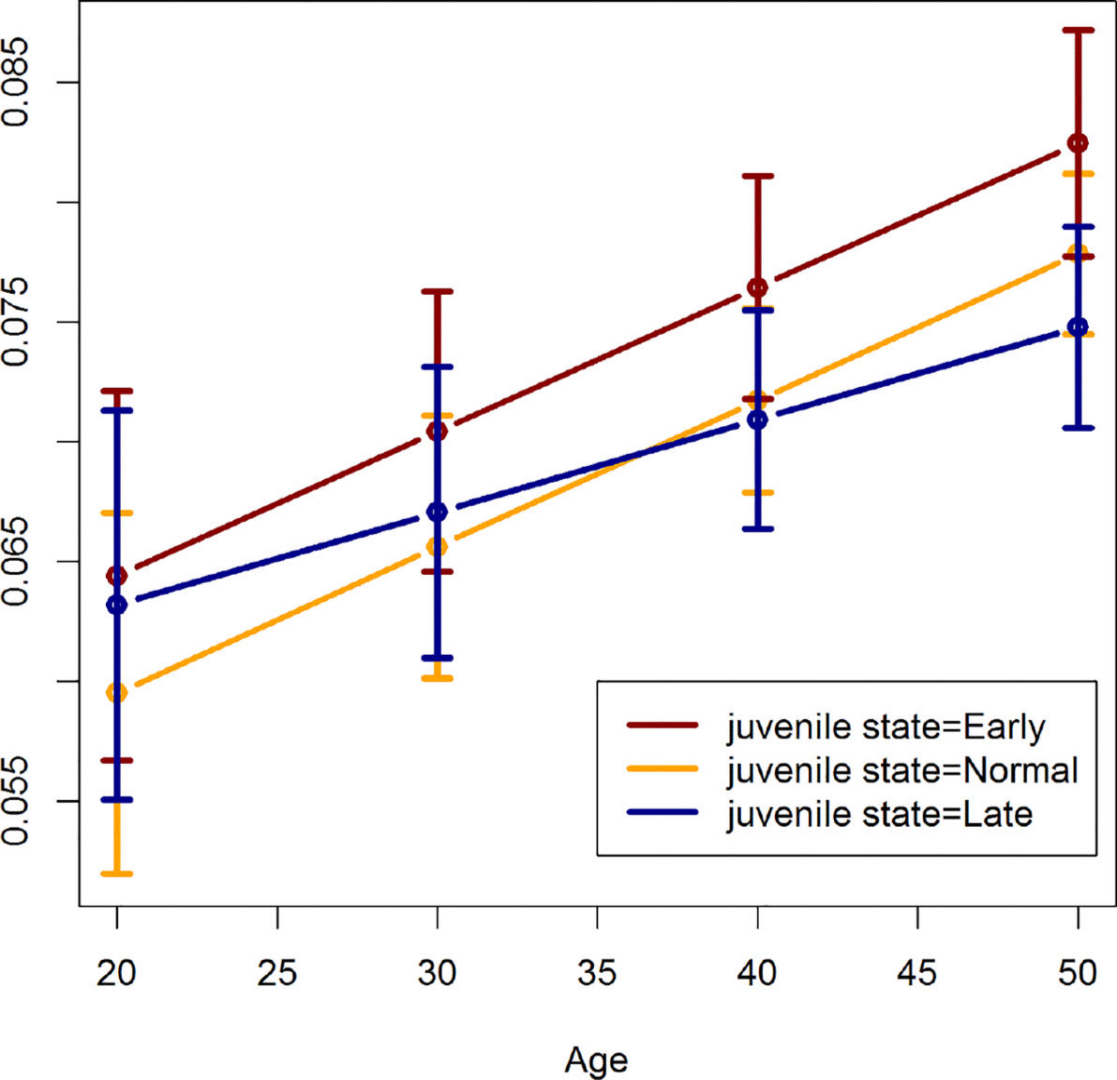
Estimated relative wall thickness index in females by juvenile state.

In males there are no significant differences in estimated RWTI between juvenile states. For females, estimated RWTI values are shown in Table 2 and Figure 4. An increasing trend of RWTI with an increasing age is clearly visible in each group.

The difference in expected RWTI between Early and Late juvenile states increases from 20 to 50 years, with the difference being significant only at 50 years. There are no significant differences in expected RWTI at any age between Normal and both Early and Late juvenile states.

### Left ventricular ejection fraction and left ventricular shortening fraction

**Figure 5:**
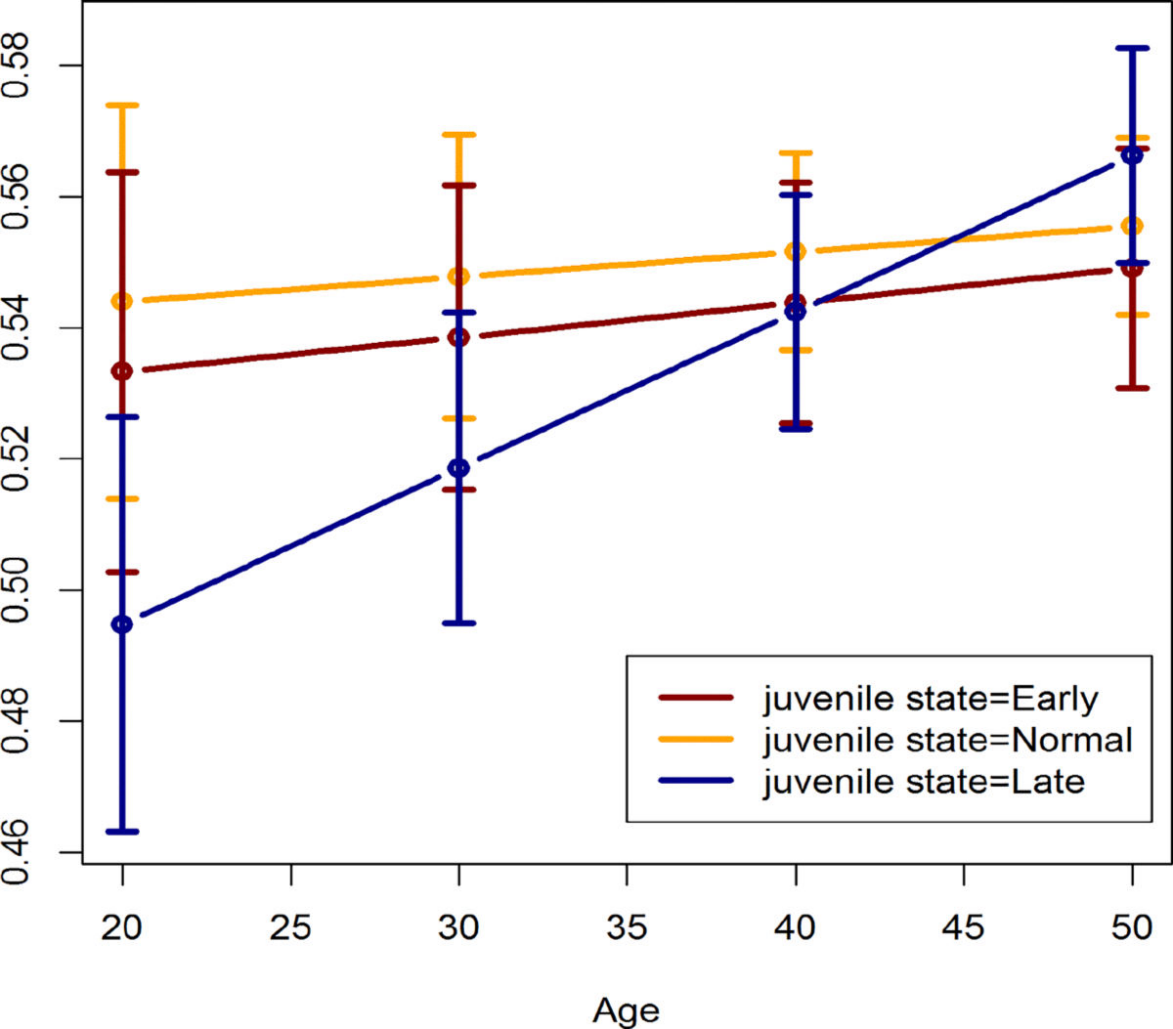
Estimated left ventricular ejection fraction in females by juvenile state.

In males we do not observe significant differences in either LVEF or LVSF between juvenile states. In contrast, we observe a significant difference in LVEF among females while there is no significant difference in LVSF between juvenile states. Unlike the previous significant differences among cardiac structure measurements, however, we observe a significant difference between Normal and Late states, instead of Early and Late (between Early and Late is not significant), in estimated LVEF at age 20 among females (p-value=0.027). At other ages, estimated LVEF values are not significantly different between juvenile states (Figure 5).

At ages 20 and 30, ordering of estimated LVEF between juvenile states is seemingly (Late, Early, Normal), which is a departure from a usual ordering of (Late, Normal, Early) in different cardiac measurements (Figures 1–4). Note that Table 2 contains test results for contrasts between Early and Late, not showing the contrast between Normal and Late.

## Discussion

The results presented here indicate that a curtailed juvenile state is associated with some abnormally high cardiac structure measurements compared to those with normal or late juvenile state. The juvenile state classification is defined on the timing of early childhood BMI rebound, implying that adult cardiac structure abnormalities may in part have their genesis in early childhood. A unique contribution of this study is to demonstrate a long-term adverse effect of the curtailed juvenile state on the adult cardiac structure and function using the FLS longitudinal data. This finding can stimulate more studies on improving cardiac health by devising an early intervention in childhood. By the way, we believe that the high quality of FLS allows us to elucidate a subtle effect of the curtailed juvenile state on cardiac structure and function decades later in life.

In this study we define a juvenile state variable (categorical) with three levels (Early, Normal, Late), based on the first and the third quartiles of the individual’s estimated age of adiposity rebound, which is originally continuous. Although it is convenient, this procedure does not take any intrinsic groupings into account. For example, there is no good justification on the number of levels (three in this study) or the critical values for different categories (the first and the third quartiles). A clustering analysis may be considered to find intrinsic categories instead of an automatic classification. We defend our procedure based on the fact that defining this categorical variable is a conservative method to model a functional relationship when the exact functional form is unknown. More studies may be needed to derive a better way of defining a categorical variable or to seek a suitable transformation of a continuous variable, at the presence of covariates, to improve power of the tests.

Since the study participants were mostly white, we do not claim that the findings of this study are applicable to other races. Due to limited measurements, we also did not account for the possibly confounding effects of lifestyle characteristics, such as nutrition, smoking, alcohol consumption, and physical activity levels. However, the insight from this study can shed light on the relationships between a curtailed juvenile state and cardiac structure and function in adulthood.

In the literature, significant associations between BMI and cardiac structure have been reported [2–7]. Obesity is also linked to various cardiovascular diseases [37,38]. The timing of BMI rebound at childhood was reported to have a strong effect on adult obesity and adult lifestyle variable [39]. Particularly, childhood obesity appears to alter cardiac structure [20–24]. Since an intervention study showed a significant improvement in cardiovascular dysfunction [40], we may hypothesize that an early intervention prior to entering a juvenile state may significantly improve cardiac structure in adulthood. A possible intervention by parents or physicians might be a nutritional change or infant breastfeeding to prevent an early attainment of adiposity rebound. Another possibility is to encourage more physical activities to limit BMI growth. More studies can elucidate which early interventions may improve health conditions later in life.

## Supplementary Material

Kim et al supplementary material

## Figures and Tables

**Table 1:** Summary statistics for measurements.

**Males**					
**measure**	**no. of obs**	**mean**	**std dev**	**min**	**max**
LVMI	273	29.8	8.7	15.2	74.2
IVSTI	273	0.17	0.04	0.1	0.34
RWTI	273	0.06	0.01	0.04	0.12
LVEF	293	0.55	0.08	0.31	0.8
LVSF	276	0.33	0.06	0.15	0.48
AGE	295	44.8	15.6	20	76.6
**Females**					
**measure**	**no. of obs**	**mean**	**std dev**	**min**	**max**
LVMI	318	28.8	8.5	12	75.1
IVSTI	318	0.2	0.05	0.12	0.62
RWTI	318	0.08	0.02	0.04	0.16
LVEF	320	0.55	0.07	0.35	0.75
LVSF	319	0.34	0.06	0.19	0.54
AGE	328	47.2	15.7	20	77.3

**Table 2:** Test results.

**Males**								
	**contrast**	**estimate**	**std error**	**DF**	**t value**	**p value**	**95% CI lower**	**95% CI upper**
LVMI	Early-Late @age 20	6.99	2.94	117	2.38	0.019	1.17	12.82
	Early-Late @age 30	5.61	2.12	117	2.65	0.009	1.41	9.81
	Early-Late @age 40	4.23	1.7	117	2.49	0.014	0.87	7.59
	Early-Late @age 50	2.85	1.95	117	1.46	0.147	−1.02	6.71
IVSTI	Early-Late @age 20	0.02	0.01	117	1.7	0.091	0	0.05
	Early-Late @age 30	0.02	0.01	117	1.78	0.077	0	0.03
	Early-Late @age 40	0.01	0.01	117	1.49	0.138	0	0.03
	Early-Late @age 50	0.01	0.01	117	0.65	0.515	−0.01	0.02
RWTI	Early-Late @age 20	0.0049	0.0049	117	0.99	0.324	−0.0049	0.0147
	Early-Late @age 30	0.0028	0.0035	117	0.8	0.425	−0.0042	0.0099
	Early-Late @age 40	0.0008	0.0028	117	0.28	0.78	−0.0048	0.0064
	Early-Late @age 50	−0.0013	0.0033	117	−0.38	0.702	−0.0077	0.0052
LVEF	Early-Late @age 20	0.0102	0.0267	128	0.38	0.704	−0.0426	0.0629
	Early-Late @age 30	0.0081	0.0197	128	0.41	0.683	−0.0309	0.047
	Early-Late @age 40	0.0059	0.0155	128	0.38	0.701	−0.0247	0.0366
	Early-Late @age 50	0.0038	0.0164	128	0.23	0.816	−0.0287	0.0364
LVSF	Early-Late @age 20	−0.0095	0.0172	120	−0.55	0.583	−0.0436	0.0247
	Early-Late @age 30	−0.0134	0.0123	120	−1.09	0.278	−0.0378	0.0109
	Early-Late @age 40	−0.0173	0.0098	120	−1.77	0.079	−0.0367	0.002
	Early-Late @age 50	−0.0213	0.0114	120	−1.86	0.065	−0.0439	0.0014
**Females**								
	**contrast**	**estimate**	**std error**	**DF**	**t value**	**p value**	**95% CI lower**	**95% CI upper**
LVMI	Early-Late @age 20	2.86	2.75	138	1.04	0.3	−2.58	8.3
	Early-Late @age 30	3.38	2.07	138	1.63	0.105	−0.71	7.48
	Early-Late @age 40	3.9	1.61	138	2.43	0.017	0.72	7.08
	Early-Late @age 50	4.42	1.57	138	2.82	0.006	1.32	7.52
IVSTI	Early-Late @age 20	0.0173	0.0147	138	1.17	0.242	−0.0118	0.0463
	Early-Late @age 30	0.0175	0.0111	138	1.58	0.116	−0.0044	0.0394
	Early-Late @age 40	0.0178	0.0085	138	2.08	0.04	0.0009	0.0347
	Early-Late @age 50	0.018	0.0083	138	2.18	0.031	0.0017	0.0344
RWTI	Early-Late @age 20	0.0012	0.0057	138	0.22	0.83	−0.01	0.0124
	Early-Late @age 30	0.0034	0.0043	138	0.79	0.431	−0.0051	0.0118
	Early-Late @age 40	0.0055	0.0033	138	1.67	0.097	−0.001	0.0121
	Early-Late @age 50	0.0077	0.0032	138	2.39	0.018	0.0013	0.014
LVEF	Early-Late @age 20	0.0385	0.0223	143	1.73	0.086	−0.0055	0.0826
	Early-Late @age 30	0.02	0.0168	143	1.19	0.237	−0.0133	0.0532
	Early-Late @age 40	0.0014	0.013	143	0.11	0.916	−0.0243	0.0271
	Early-Late @age 50	−0.0172	0.0124	143	−1.39	0.168	−0.0418	0.0073
LVSF	Early-Late @age 20	0.0151	0.0156	139	0.97	0.334	−0.0157	0.046
	Early-Late @age 30	0.0101	0.0118	139	0.86	0.391	−0.0132	0.0334
	Early-Late @age 40	0.0051	0.0091	139	0.57	0.573	−0.0128	0.023
	Early-Late @age 50	0.0001	0.0086	139	0.01	0.99	−0.0169	0.0171
