# Supplementary material for: Effects of Curtailed Juvenile State on Cardiac Structure and Function in Adulthood: The Fels Longitudinal Study
Source: J Child Obes. Author manuscript; Available in PMC 2020 Jun 4. (PMC7271876; doi:10.21767/2572-5394.100018)
Supplement: Kim et al supplementary material [file NIHMS1590623-supplement-Kim_et_al_supplementary_material.pdf]

Supplementary material

Effects of Curtailed Juvenile State on Cardiac Structure and Function in Adulthood: The Fels Longitudinal Study

Nak-Kyeong Kim, Roy T. Sabo, Aobo Wang, Cynthia S. Sabo, and Shumei S. Sun

Table S1. Parameter estimates

"State" variable can take one of three levels, (-1, 0, 1) = (Early, Normal, Late). In this table, State level 1 is a reference level, which does not have a corresponding parameter estimate.

Males

	effect	State	estimate	std error	DF	t value	p value
**LVMI**	Intercept		14.454	3.518	150	4.11	<.0001
	State	-1	9.760	4.955	150	1.97	0.0507
	State	0	2.312	4.395	150	0.53	0.5996
	Age		0.317	0.086	117	3.69	0.0003
	Age*State	-1	-0.138	0.113	117	-1.23	0.2218
	Age*State	0	-0.015	0.101	117	-0.15	0.8797
**IVSTI**	Intercept		0.10300	0.01539	150	6.69	<.0001
	State	-1	0.03279	0.02173	150	1.51	0.1334
	State	0	0.00694	0.01922	150	0.36	0.7185
	Age		0.00145	0.00038	117	3.85	0.0002
	Age*State	-1	-0.00054	0.00050	117	-1.10	0.2739
	Age*State	0	-0.00005	0.00044	117	-0.11	0.9160
**RWTI**	Intercept		0.04171	0.00591	150	7.05	<.0001
	State	-1	0.00899	0.00835	150	1.08	0.2834
	State	0	-0.00009	0.00738	150	-0.01	0.9905
	Age		0.00044	0.00015	117	3.06	0.0028
	Age*State	-1	-0.00020	0.00019	117	-1.08	0.2839
	Age*State	0	0.00002	0.00017	117	0.09	0.9263
**LVEF**	Intercept		0.50340	0.02946	159	17.08	<.0001
	State	-1	0.01437	0.04353	159	0.33	0.7417
	State	0	-0.03180	0.03813	159	-0.83	0.4055
	Age		0.00095	0.00068	128	1.40	0.1629
	Age*State	-1	-0.00021	0.00094	128	-0.22	0.8236
	Age*State	0	0.00073	0.00084	128	0.87	0.3853
**LVSF**	Intercept		0.30560	0.02029	150	15.06	<.0001
	State	-1	-0.00162	0.02932	150	-0.06	0.9560
	State	0	-0.01077	0.02562	150	-0.42	0.6749
	Age		0.00071	0.00050	120	1.41	0.1601
	Age*State	-1	-0.00039	0.00067	120	-0.58	0.5598
	Age*State	0	-0.00001	0.00060	120	-0.02	0.9829

Females

	effect	State	estimate	std error	DF	t value	p value
**LVMI**	Intercept		18.614	3.137	174	5.93	<.0001
	State	-1	1.821	4.347	174	0.42	0.6757
	State	0	-1.898	4.297	174	-0.44	0.6593
	Age		0.172	0.062	138	2.75	0.0067
	Age*State	-1	0.052	0.089	138	0.59	0.5595
	Age*State	0	0.084	0.085	138	0.99	0.3239
**IVSTI**	Intercept		0.11780	0.01667	174	7.07	<.0001
	State	-1	0.01676	0.02318	174	0.72	0.4706
	State	0	0.00567	0.02298	174	0.25	0.8053
	Age		0.00150	0.00033	138	4.51	<.0001
	Age*State	-1	0.00003	0.00047	138	0.05	0.9573
	Age*State	0	-0.00005	0.00046	138	-0.10	0.9186
**RWTI**	Intercept		0.05546	0.00645	174	8.60	<.0001
	State	-1	-0.00307	0.00896	174	-0.34	0.7322
	State	0	-0.00814	0.00887	174	-0.92	0.3597
	Age		0.00039	0.00013	138	3.01	0.0031
	Age*State	-1	0.00022	0.00018	138	1.17	0.2422
	Age*State	0	0.00022	0.00018	138	1.27	0.2074
**LVEF**	Intercept		0.44700	0.02508	171	17.82	<.0001
	State	-1	0.07571	0.03492	171	2.17	0.0315
	State	0	0.08932	0.03515	171	2.54	0.0119
	Age		0.00239	0.00050	143	4.80	<.0001
	Age*State	-1	-0.00186	0.00070	143	-2.64	0.0092
	Age*State	0	-0.00200	0.00071	143	-2.84	0.0052
**LVSF**	Intercept		0.27900	0.01745	174	15.98	<.0001
	State	-1	0.02517	0.02448	174	1.03	0.3053
	State	0	0.01539	0.02421	174	0.64	0.5259
	Age		0.00135	0.00035	139	3.91	0.0001
	Age*State	-1	-0.00050	0.00049	139	-1.02	0.3096
	Age*State	0	-0.00038	0.00049	139	-0.77	0.4431
